# Refinement and calibration of a human PBPK model for the plasticiser, Di-(2-propylheptyl) phthalate (DPHP) using *in silico*, *in vitro* and human biomonitoring data

**DOI:** 10.3389/fphar.2023.1111433

**Published:** 2023-02-02

**Authors:** Kevin McNally, George Loizou

**Affiliations:** Health and Safety Executive, Buxton, United Kingdom

**Keywords:** plasticiser, DPHP, PBPK, biomonitoring, reverse dosimetry, *in silico*

## Abstract

An existing physiologically based pharmacokinetic model for Di-(2-propylheptyl) phthalate (DPHP) was refined to improve the simulations of the venous blood concentrations of the primary monoester metabolite, mono-(2-propylheptyl) phthalate (MPHP). This was considered a significant deficiency that should be addressed because the primary metabolite of other high molecular weight phthalates has been associated with toxicity. The various processes that influence the concentration of DPHP and MPHP in blood were re-evaluated and modified. A few simplifications of the existing model were made, including the removal of enterohepatic recirculation (EHR) of MPHP. However, the primary development was describing the partial binding of MPHP to plasma proteins following uptake of DPHP and metabolism in the gut affording better simulation of the trends observed in the biological monitoring data. Secondly, the relationship between blood concentrations and the urinary excretion of secondary metabolites was explored further because the availability of two data streams provides a better understanding of the kinetics than reliance on just one. Most human studies are conducted with few volunteers and generally with the absence of blood metabolite measurements which would likely imply an incomplete understanding of the kinetics. This has important implications for the “read across” approach proposed as part of the development of New Approach Methods for the replacement of animals in chemical safety assessments. This is where the endpoint of a target chemical is predicted by using data for the same endpoint from another more “data rich” source chemical. Validation of a model parameterized entirely with *in vitro* and *in silico* derived parameters and calibrated against several data streams would constitute a data rich source chemical and afford more confidence for future evaluations of other similar chemicals using the read-across approach.

## 1 Introduction

Plasticisers are different classes of chemicals used in the manufacture of plastics to create products of varying flexibilities and brittleness. Phthalates, which are some of the most used plasticisers, are dialkyl- or alkylarylesters of 1, 2-benzenedicarboxylic acid. The length of the ester chain determines the industrial application, with alkyl chain lengths from three to 13 carbons widely used in polymers such as polyvinyl chloride (PVC).

Di-(2-propylheptyl) phthalate (DPHP), CAS No. 53306-54-0, marketed under the trade name Palatinol^®^10-P, is a high molecular weight branched phthalate ester which is used in the manufacture of PVC products. While DPHP is predominantly recommended for technical applications, and has in the past been found in toys, food packaging and medicinal products (Klein et al., 2018) the European Union has advised against its use as well as not providing clearance for use in food contact materials. Several studies have demonstrated the presence of DPHP in the general population (Wittassek and Angerer 2008; Wittassek et al., 2011; Schutze et al., 2015; Schmidtkunz et al., 2019; Schwedler et al., 2019).

Adverse effects observed with other phthalates that were related to metabolism of the parent phthalate to the primary monoester are not reported to occur with DPHP (Oishi and Hiraga 1980; Foster et al., 1981; Sjöberg et al., 1986). However, a risk estimation for DPHP centred on its primary monoester metabolite, mono-(2-propylheptyl) phthalate (MPHP) was proposed by Klein et al. (2018) based on the large variations in species-specific burdens observed in venous blood concentrations of mono(2-ethylhexyl)phthalate (MEHP), the primary monoester of di-(2-ethylhexyl) phthalate (DEHP) (Rhodes et al., 1986; Kessler et al., 2004; Kurata et al., 2012). A study involving the biological monitoring (BM) of human volunteers following administration of a single oral dose of DPHP was conducted for the purpose of estimating the risk of DPHP (Klein et al., 2018).

**FIGURE 1:**
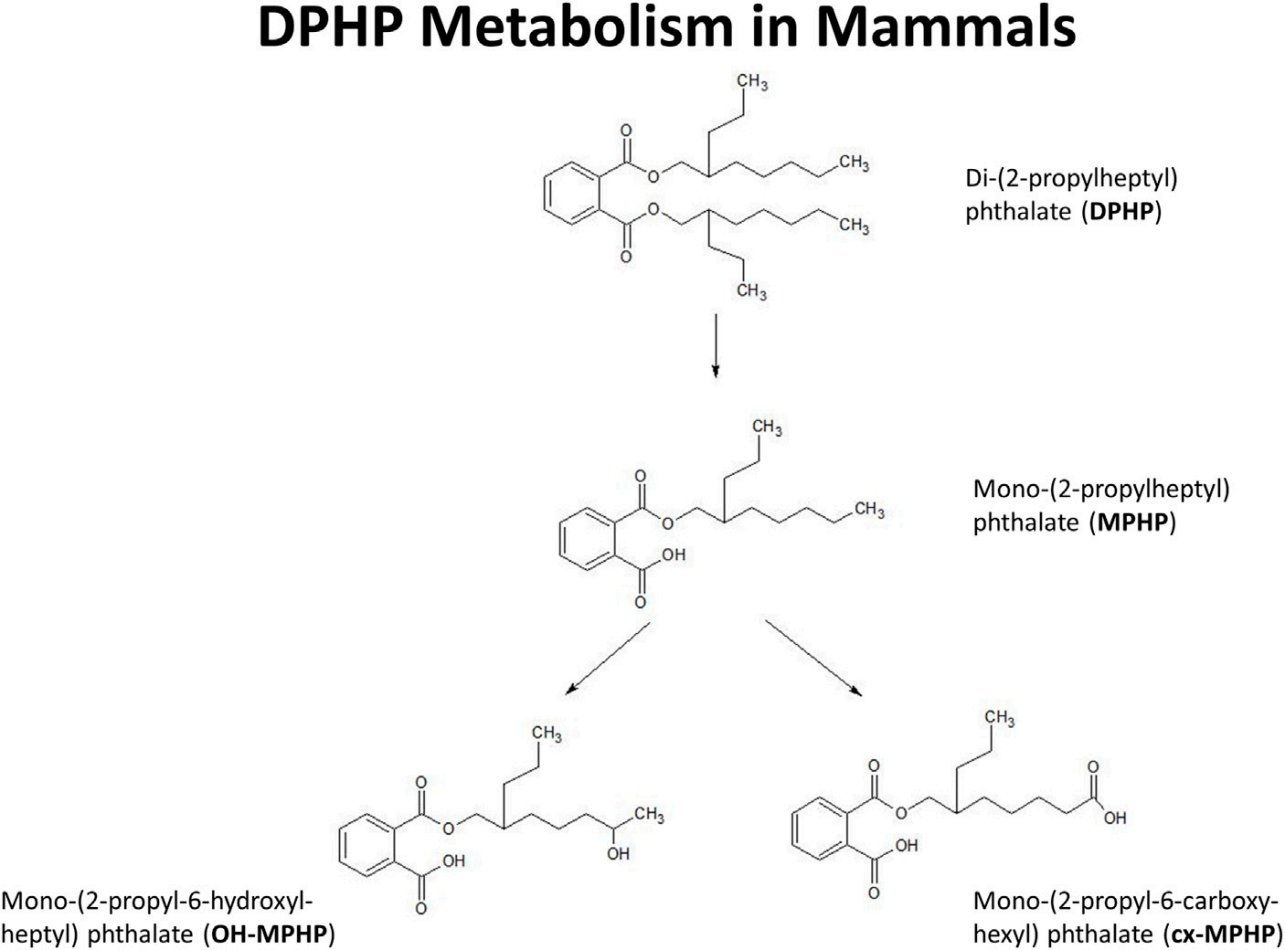
Postulated metabolism of DPHP in humans showing only those metabolites measured in human biological monitoring and described in the PBPK model.

In this study we present a refinement of the PBPK model for DPHP developed previously (McNally et al., 2021) to interpret the BM data from the study of Klein et al. (2018). These were, venous blood concentrations of DPHP and its primary monoester metabolite, MPHP, and the urinary excretion of MPHP and the two direct, secondary metabolites of MPHP, mono-(2-propyl-6-hydroxyheptyl) phthalate (OH-MPHP) and mono-(2-propyl-6-carboxyhexyl) phthalate (cx-MPHP) (Figure 1).

The primary motivation for the refinement of the existing PBPK model for DPHP was that the fits to MPHP in venous blood were poor, with the PBPK model significantly underestimating measured concentrations. Given that the first metabolite of other high molecular weight phthalates has been associated with toxicity this was considered to be an important deficiency in the model that we sought to resolve by re-evaluating the various processes that influence the concentration of DPHP and MPHP in blood. Secondly, we sought to further explore the relationship between blood concentrations and the urinary excretion of secondary metabolites because the availability of two data streams provides a better understanding of the kinetics than reliance on just one. Human studies are conducted with few volunteers and generally with the absence of blood metabolite measurements which would likely imply an incomplete understanding of the kinetics. Validation of a model based on two data streams should afford more confidence for future evaluations of other similar chemicals using a read-across approach. Thirdly, we assess inter- and intra-species differences and comment on the assumptions of extrapolation from rat to human.

## 2 Materials and methods

### 2.1 The PBPK model

**FIGURE 2:**
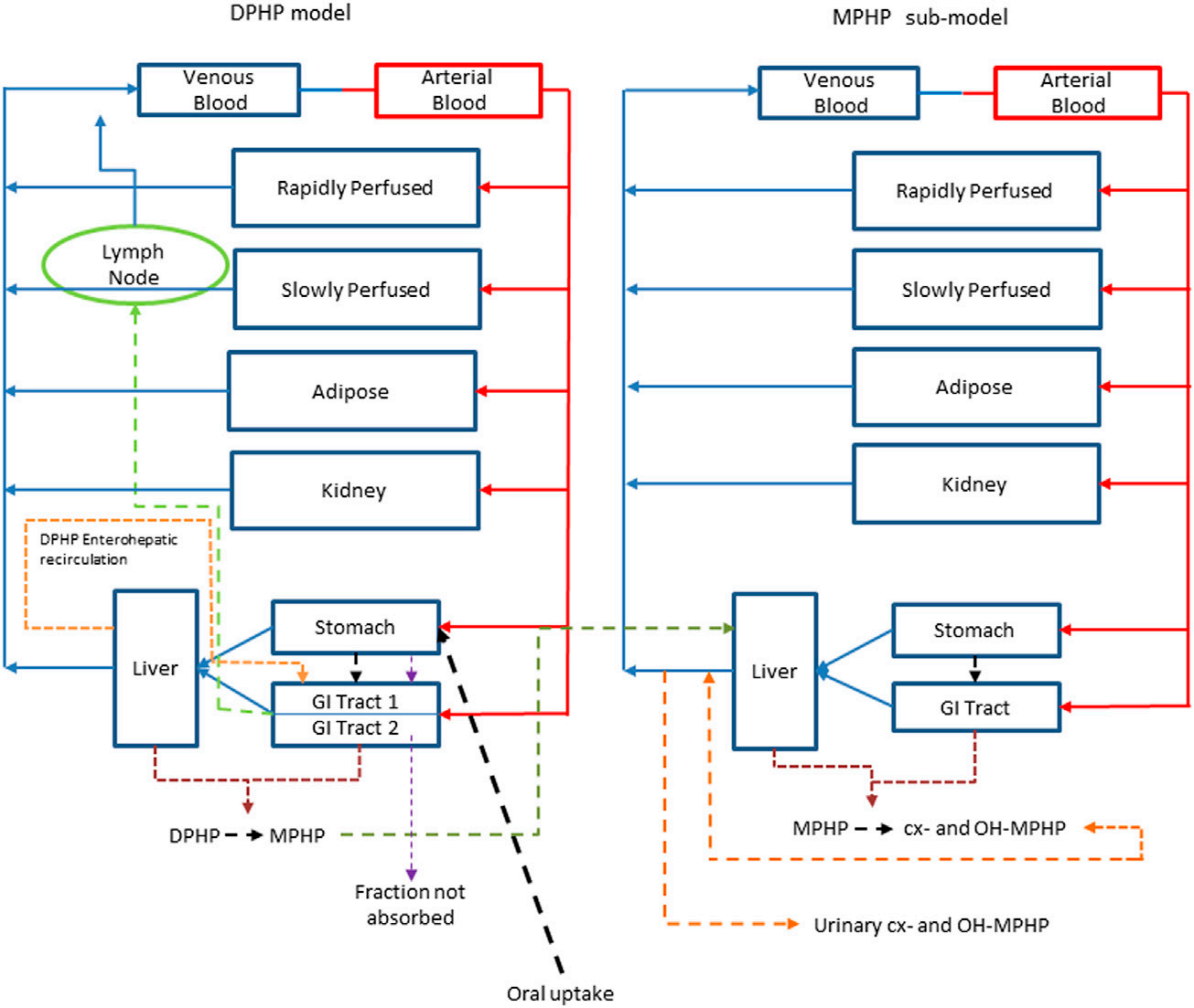
A schematic of the model for DPHP and sub-model for MPHP. The main model contained a lymphatic compartment (- - - -) which received a portion of oral dose from the stomach and GI tract. Urinary excretion of metabolites was described with a first-order elimination rate constant ascribed to the sub-model.

An existing human PBPK model for DPHP (McNally et al., 2021) was modified further to study the absorption, distribution, metabolism, and elimination of DPHP following single oral doses. The key changes compared with McNally et al. (2021) were the inclusion of a kidney compartment so that an additional dataset of MPHP in urine could be utilised in model calibration, a modification to the uptake of MPHP from the gut to facilitate an improved fit to DPHP and MPHP in blood, and in the absence of Michaelis-Menten constants, a simple modification to the metabolism of MPHP, specified using clearance, to account for a limitation of metabolism, whose mechanism is uncertain, perhaps saturation or inhibition of metabolism, thus allowing a fraction of MPHP created in the liver through metabolism of DPHP to escape into blood. Furthermore, a few simplifications of the model structure were made where data indicated this was appropriate (Figure 2).

**FIGURE 3:**
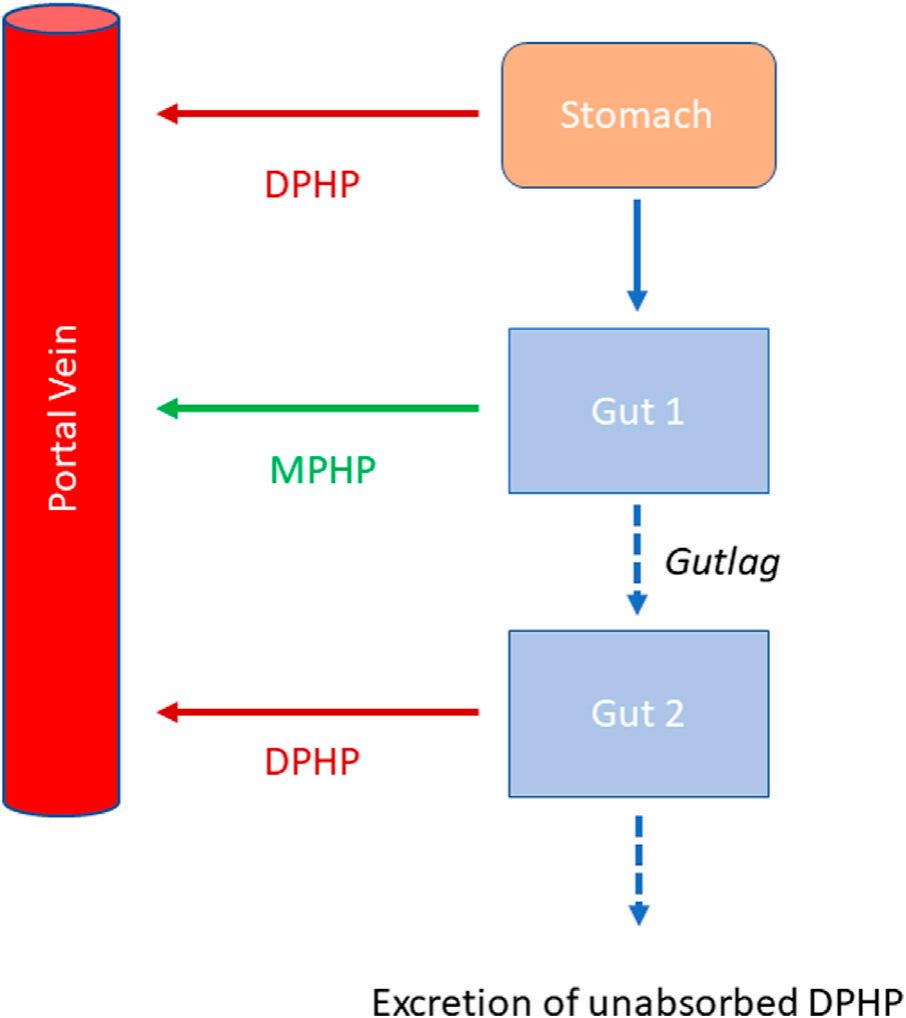
Schema of the modified stomach and gut sub model showing the introduction of DPHP into the stomach *via* oral administration, transport of DPHP from the stomach to the GI tract with metabolism of DPHP to MPHP in gut Section 1 and the delay term, *Gutlag* controlling the transport of DPHP into gut Section 2.

Briefly, the model for DPHP described two distinct uptake processes and allowed for a majority fraction to pass directly through the gut and be ultimately eliminated in faeces. The primary uptake process was into blood. The model included a description of absorption from the stomach and gastro-intestinal (GI) tract, simplified to a two-stage intestine compartment (Figure 3). Uptake of DPHP into venous blood from the stomach, metabolism of DPHP to MPHP and uptake of MPHP into venous blood was ascribed to the first gut compartment, and finally uptake of DPHP into venous blood was ascribed to the second-phase gut-compartment. A parameter, *Gutlag* was included to represent a delay in transport of DPHP through the gut transiting from the first to second gut compartment. An important change compared with McNally et al. (2021) was that a fraction of MPHP absorbed through the first gut compartment was unavailable for first pass metabolism in the liver following uptake into venous blood—this represents incomplete binding. The second uptake mechanism of DPHP was into the lymphatic system. Uptake of DPHP *via* the lacteals in the intestine and entering venous blood after bypassing the liver was coded. Inclusion of a lymph compartment was based on the assumption that DPHP, like DEHP, binds like lipid to lipoproteins (Griffiths et al., 1988) which are formed in enterocytes and transported in the lymph to enter the venous blood *via* the thoracic duct (Kessler et al., 2012). The fractions of dose entering venous blood, the lymphatic system and passing straight through the gut summed to unity.

Metabolism of DPHP to MPHP was ascribed to the liver and a section of the gut (Figure 3, gut 1). The model for DPHP additionally encoded the transport process of enterohepatic recirculation. Uptake of DPHP from the liver into bile was modelled as a first order uptake process with a delay (to represent transport from liver to gut) before DPHP appeared in the small intestine where DPHP was available for reabsorption.

The model had a stomach and (the two-phase intestine) draining into the liver and systemically circulated to adipose, kidney, blood (plasma and red blood cell) and slowly and rapidly perfused compartments (Figure 2). Protein binding was described in arterial blood, with only the unbound fraction of DPHP available for distribution to organs and tissues and metabolism.

A sub-model was coded to describe the kinetics of MPHP. As described above, metabolism of DPHP to MPHP was coded in the first intestinal compartment and the liver, therefore models for DPHP and MPHP were connected at these nodes in the model. Metabolism of MPHP was coded in the liver alone. A fraction of MPHP (Escapeli) was coded as unavailable for immediate metabolism: in the absence of *in vitro* determined Michaelis-Menten constants this is a simple modification to the clearance-based first-order model for metabolism to the represent saturation or inhibition of metabolism. The MPHP sub-model had a stomach and (single-phase) intestine draining into the liver and systemically circulated to adipose, kidney, blood (plasma and red blood cell) and slowly and rapidly perfused compartments (Figure 2). Elimination of MPHP from the kidney was described with a first-order elimination rate; this represents a modification compared to McNally et al. (2021). Furthermore, in contrast to the previously published model, enterohepatic recirculation of MPHP was not considered. As with the DPHP model, binding was described in arterial blood.

To make use of biological monitoring data on two metabolites of MPHP (OH-MPHP and cx-MPHP) it was necessary to include the elimination of these substances into urine. A simplified representation of these downstream metabolites was assessed as being suitable for the aims of modelling. The appearance of OH-MPHP and cx-MPHP in blood were coded as respective fractions of metabolised MPHP. First order elimination constants described the removal of second order metabolites, OH-MPHP and cx-MPHP from blood into the urine. The model did not describe the distribution of these metabolites to organs and tissues.

The model code is available in Supplementary Materials.

### 2.2 Baseline parameterisation

Anatomical, physiological, physicochemical, and metabolic parameters were very similar to the previous model for DPHP (McNally et al., 2021) with small differences arising due to changes to model structure. These parameters are provided in Tables 1–3. Sources, derivation and calculation of parameters are described in Supplementary Materials.

**TABLE 1:** Tissue:blood partition coefficients and plasma fraction unbound predicted using Log P_ow_.

	DPHP	MPHP
Log Po:w	10.83	5.3
Tissue:blood partition coefficient		
Plasma	15.5	25.23
Adipose	63.4	29.10
Liver	5.89	54.8
Kidney	5.89	15.5
Muscle	3.29	7.51
Red blood cells	3.01	6.67
Gut	7.4	25.2
Spleen	3.7	12.20
Stomach^a^ (gut)	7.4	25.2
Rapidly Perfused (spleen)	3.7	12.20
Slowly Perfused (muscle)	3.29	7.51
Plasma Fraction Unbound	0.0025	0.0146

^a^ Compartments in italics have surrogate values from another organ compartment. The corresponding surrogate organ compartment is in parentheses.

**TABLE 2:** Volunteer specific parameters.

	Volunteers					
	A	B	C	D	E	F
Body weight (kg)	83	75	76	74	90	108
Dose (mg kg−1)	0.717	0.639	0.781	0.783	0.775	0.733
Fraction Metabolised						
FracMetab to cx_MPHP	0.02	0.017	0.02	0.023	0.018	0.018
FracMetab to OH_MPHP	0.396	0.340	0.374	0.359	0.334	0.329

**TABLE 3:** Physiological and kinetic default values used in PBPK model and probability distributions applied for uncertainty and sensitivity analyses.

Physiological parameters	Abbreviation	Default value	Distribution
Body weight (kg)	BW	72.3	N^a^(72.3, 9.05)
% BW			
Total vascularised tissues	VT	0.95	-
Liver	VLiC	3.09	N(3.09, 0.8)
Kidney	VKic	0.58	N(0.58, 0.15)
Fat	VFaC	19.5	LN(3.42, 0.43)
Gut	VGuC	1.50	N(1.5, 0.2)
Stomach	VStC	0.22	N(0.22, 0.07)
Slowly perfused tissue	VSpdC	60.7	N(60.7, 9.4)
Rapidly perfused tissue	VRpdC	3.71	N(3.7, 0.26)
Blood	VBldC	5.0	N(5.0, 1.0)
Cardiac output (L h^-1^ kg^-1^ BW)	QCC	14	N(13.8, 2.5)
% Cardiac output			
Liver	QHepartC	6.9	N(6.9, 0.5)
Kidney	QKiC	20	N(20.0, 3.0)
Fat	QFaC	5.0	N(5.3, 0.3)
Gut	QGuC	14.9	N(14.9, 3.0)
Stomach	QStC	1.1	N(1.1, 0.1)
Slowly perfused tissue	QSpdC	28.7	N(28.7, 1.9)
Rapidly perfused tissue	QRpdC	23.0	N(23.0, 2.8)
Metabolic Clearance (minutes)			
In vitro half-life DPHP	T_½DPHP_	3^b^	HN(10)
In vitro half-life MPHP	T_½MPHP_	8.05	N(8.05, 4.0)
In vivo DPHP gut half-life	T_½DPHP_gut_	60^c^	N(30, 10)
Protein Binding (%)			
DPHP	FB_DPHP	99.75	U(0.95, 1.00)
MPHP	FB_MPHP	98.54	U(0.90, 1.00)
Elimination (liver to bile) (h^-1^)			
DPHP	k1_DPHP_liver	10	HN(10)
Microsomal protein yield (mg g^-1^)			
Hepatic	MPY	34^d^	N(34, 10)
Gut	MPY_gut_	3.9^e^	N(3.9, 2.0)

Parameters	Abbreviation	Default value	Distribution
Blood:tissue partition coefficients			
*DPHP*			
Plasma	Pbab	15.5	U(1, 30)
Adipose	Pfab	63.4	U(32, 125)
Liver	Plib	5.89	U(1, 50)
Kidney	Pkib	5.89	U(3, 12)
Red blood cells	Prbcb	3.29	U(3, 12)
Gut	Pgub	7.4	U(1, 50)
Stomach	Pstb	3.7	U(2, 8)
Rapidly Perfused	Prpdb	3.7	U(2, 8)
Slowly Perfused	Pspdb	3.29	U(2, 8)
*MPHP*			
Plasma	PbaM	25.23	U(1, 50)
Adipose	PfaM	29.10	U(15, 60)
Liver	PliM	54.8	U(1, 30)
Kidney	PkiM	15.5	U(1, 30)
Red blood cells	PrbcM	6.67	U(3, 12)
Gut	PguM	25.2	U(1, 30)
Stomach	PstM	25.2	U(12, 50)
Rapidly Perfused	PrpdM	12.20	U(6, 24)
Slowly Perfused	PspdM	7.51	U(4, 15)
Gastric emptying (h^-1^)^f^			
Maximum	k_(max)_	10.2	U(5.1, 20.4)
Minimum	k_(min)_	0.005	U(0.0025, 0.01)
Absorption (fraction of dose)			
Fraction of dose taken up into liver	FRACDOSEHep	0.1	U(0.001, 0.4)
Fraction of dose taken up into lymph	FRACDOSELymph	0.5	U(0.001, 0.15)
Absorption (h^-1^)			
Gut	k_Ga_	25.1	U(12.55, 50.2)
Absorption in Stomach	BELLYPERM	0.685	U(0.05, 7.5)
Absorption in first GI Tract compartment	GIPERM1	5.1	U(0.05, 10)
Absorption in first GI Tract compartment	GIPERM2	5.1	U(0.05, 30)
Absorption in Lymph via stomach	BELLYPERMLymph	0.685	U(0.34, 0.99)
Absorption in Lymph via GI Tract	GIPERMLymph	5.1	U(2.6, 7.6)
Absorption into blood from lymph	K1Lymph	0.2	U(0.05, 3)
Delays (h^-1^)			
Transportation delay in gut	Gutlag	1	U(0.01, 5)
Delay associated with lymphatic uptake	LymphLag	1	(0.01, 7)
Fraction unavailable for first pass metabolism (fraction of dose)			
MPHP in gut	EscapeFracgut	0.5	U(0, 1)
MPHP in liver	EscapeFracliver	0.5	U(0, 1)
Metabolism of MPHP (fractions)			
Fraction of MPHP to OH-MPHP	FracMetabOH	0.3	U(0.2, 0.5)
Fraction of MPHP to cx-MPHP	FracMetabcx	0.02	U(0.01, 0.05)
Urinary elimination rate (h^-1^)			
5OH-MEHA	K1_MOH	1	U(0.01, 5)
5cx-MEPA	K1_5cx	1	U(0.01, 5)

^a^ Distributions, N, normal; LN, Lognormal; HN, Half normal; U, uniform.

^b^ Estimated.

^c^ Estimated.

^d^ Barter et al. (2007), Howgate et al. (2006)

^e^ Pacifici, et al. (1988), Soars, et al. (2002)

^f^ Loizou and Spendiff (2004)

### 2.3 Biological monitoring data

The BM data described in (Klein et al., 2018) were kindly provided by Dr. Rainer Otter of BASF, SE. Briefly, DPHP was administered orally to six healthy male volunteers (designated A to F), aged be-tween 30 and 64 years, weighing between 74 and 108 kg. A single dose of 738 ± 56 μg/kg BW DPHP was administered as an emulsion of 7% (w/v) in an aqueous saccharose solution (70% w/v) between 45 and 140 min after breakfast. The resulting respective doses for the six individuals were between 0.639 and 0.783 mg/kg body weight (Table 2).

Klein et al. (2018) report on the trends in blood specimens of volunteers over a 24-h period following ingestion of DPHP with samples collected at 0.25-, 0.5-, 0.75-, 1-, 2-, 3-, 4-, 5-, 6-, 7-, 8-, 10- and 24-h following exposure. The total concentrations of DPHP and MPHP (nM) were extracted from the dataset. BM data were converted to units of mg/L or µg/L prior to use in calibration. Klein et al. (2018) also reported on trends of metabolites of DPHP in urine specimens over a 46-h period following ingestion, with samples collected at 1-, 2-, 3-, 4-, 5-, 6-, 8-, 10-, 12-, 14-, 18-, 22-, 26-, 30-, 34-, 38-, 42- and 46 h. The concentrations of MPHP, cx-MPHP and OH-MPHP (mg/L) were extracted from the dataset. The rates of deposition of metabolite into the bladder (mg/h) were calculated based on the concentrations (mg/L), the volume of the urine void L) and the time between successive voiding events. This rate represents an average rate of deposition since the previous urination event and renders the trends in urine data more clearly (Nehring et al., 2020). The derived rate was associated with the mid-point between the two voiding events.

**FIGURE 4:**
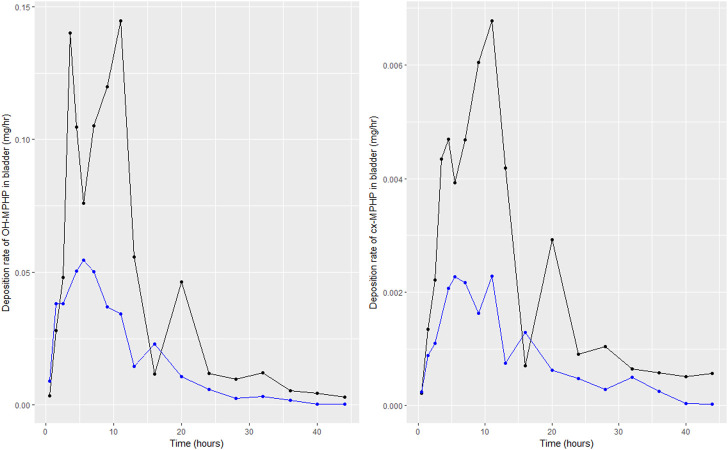
Data on rates of the deposition of OH-MPHP and cx-MPHP into the bladder, calculated for individuals C (blue line and points) and E (black line and points) from the data of Klein et al. (2018). Panel **(A)** shows the profiles of OH-MPHP; panel **(B)** shows the profiles of cx-MPHP.

Following a review of data, the BM data from volunteers C and E were inconsistent with data from the other four volunteers; for these two volunteers a second large spike at 15 and 20 h of second order metabolites (cx-MPHP and OH-MPHP) was observed in urine voids (Figure 4). This feature of the data would be consistent with a second significant uptake of DPHP from the gut associated with the intake of food/drink. An exclusion of these data was regarded by the authors as preferable to further modifications to model structure based on weak evidence. Only data from volunteers A, B, D and F were taken forward into calibration. We comment further on exclusions of data in the discussion.

## 3 Statistical analysis

### 3.1 Parameter distributions

Probability distributions for uncertainty and sensitivity analysis of the final PBPK model are listed in Table 3. Anatomical and physiological parameter distributions were obtained from the freely available web-based application PopGen (McNally et al., 2014). A population of 10,000 individuals comprising of 100% Caucasian males was generated. The range of ages, heights and body weights supplied as input to PopGen were chosen to encompass the characteristics of the volunteers who participated in the human volunteer study (Klein et al., 2018). Parameter ranges for organ masses and blood flows were modelled by normal or log-normal distributions as appropriate with parameters estimated from the sample and truncated at the 5th and 95th percentiles.

Uniform distributions were ascribed to the various delay terms and uptake and elimination rates. The upper and lower bounds in Table 3 were refined during the model development process. The tabulated values are therefore based upon expert judgement and represent conservative yet credible bounding estimates.

### 3.2 Uncertainty and sensitivity analysis

McNally et al. (2021) describe an interactive approach for development and testing of the PBPK model for DPHP using techniques for uncertainty and sensitivity analysis to study the behaviour of the model and the key parameters that drove variability in the model outputs. For this refined model a less intensive testing process was required. The key goal of this analysis was to determine whether the change to encode partial binding of MPHP in the gut was sufficient to allow the model to better fit the BM data on concentrations of MPHP in venous blood, whilst not sacrificing the quality of fit of predictions of the deposition of OH-MPHP and cx-MPHP in urine. Furthermore, the study of MPHP in urine, a new measure for use in calibration, was necessary to ensure model predictions were broadly credible. Sensitivity analysis of the concentration of MPHP in venous blood and the rate of deposition of MPHP in the bladder (mg/h) was subsequently conducted to assess whether further sensitive parameters should be taken forward to calibration.

Uncertainty analysis was conducted though rejection sampling. A 500-point maxi-min Latin Hypercube Design (LHD) based upon the probability distributions given in Table 3 was created and the PBPK model was run for each of these design points. All simulations where predictions of the peak concentration of MPHP in blood (mg/L) was less than 0.025 or greater than 0.3 mg/L and where predictions of MPHP in blood (mg/L) at 48 h were more than 0.025 mg/L were rejected. These were generous constraints based upon the trends in BM from the volunteers. The concentration response trends for MPHP in blood (mg/L), and the rate of deposition of MPHP and OH-MPHP into urine (mg/h) were studied for the retained sample to assess the credibility of the model form.

Sensitivity analysis of MPHP in blood (mg/L) and rate of deposition of MPHP into the bladder was conducted using elementary effects screening (Morris test). A total of 61 parameters were varied with five elementary effects per input computed, leading to a design of 325 runs of the PBPK model. The Morris test was applied to model output at 0.5- and 3-h following ingestion, which were broadly representative of the prior to peak concentration and post-peak concentration periods.

### 3.3 Calibration

Calibration is the process of tuning a subset of model parameters such that the discrepancy between model predictions and comparable measurement data is minimised. This is achieved through the specification of an error model that links predictions to measurements. A Bayesian approach was followed (McNally et al., 2012) for calibration in this work since this allows the uncertainty in parameters (and thus on the concentration response predictions from the PBPK model) to be explicitly quantified.

A Bayesian approach requires the specification of a joint prior distribution for the parameters under study. It is necessary to distinguish between two classes of parameters: Global parameters which are common to all individuals (appropriate for various constants and physicochemical properties such as partition coefficients etc.,); and local parameters, which vary between individuals (suitable for accounting for variability in the physiology and modelling the participant specific uptake of DPHP etc.,). These two classes are denoted by the vectors θ ; $\omega_{j}$ respectively, where the subscript *j = 1 … 4,* denotes the participant. A prior distribution for each global parameter was specified through the distributions provided in Table 3. A prior distribution for each individual (four copies in all) was specified for each of the local parameters. These distributions are also provided in Table 3. A median and 95% interval for global and local parameters is provided in Tables 4 (global) and Table 5 (locals) respectively.

**TABLE 4:** Global prior and posterior pdarameter distributions.

Parameter	Median (95% interval)	
	Prior	Posterior
FB_DPHP	0.975 (0.951, 0.999)	0.987 (0.986, 0.989)
FB_MPHP	0.950 (0.902, 0.998)	0.945 (0.922, 0.959)
DPHP_GUT_half_life	30.13 (10.67, 49.37)	42.72 (29.27, 58.21)
DPHP_half_life	6.62 (0.322, 22.34)	7.73 (5.65, 15.11)
Pbab	15.5 (1.785, 29.27)	18.17 (7.33, 29.56)
Pgub	25.23 (2.12, 48.70)	31.99 (24.11, 39.94)
Plib	25.23 (2.12, 48.70)	2.03 (1.04, 11.86)
PbaM	25.23 (2.12, 48.70)	36.86 (14.64, 49.48)
PliM	15.5 (1.785, 29.27)	6.28 (1.21, 27.55)
PguM	15.5 (1.785, 29.27)	16.89 (3.53, 28.84)
PkiM	15.5 (1.785, 29.27)	4.08 (2.18, 7.93)
K1_MPHP	2.52 (0.15, 4.87)	0.72 (0.35, 1.21)
K1_MOH	2.52 (0.15, 4.87)	2.69 (1.75, 4.59)
K1_cx	2.52 (0.15, 4.87)	1.64 (1.18, 2.42)
FracMetab_MOH	0.35 (0.21, 0.49)	0.221 (0.211, 0.242)
FracMetab_cx	0.03 (0.011, 0.049)	0.010 (0.010, 0.011)
Escape_gu	0.50 (0.028, 0.977)	0.59 (0.31, 0.83)
Escape_Li	0.50 (0.028, 0.977)	0.59 (0.45, 0.75)
$\sigma_{DPHP\_B}$	0.67 (0.032, 2.20)	0.0048 (0.0039, 0.0065)
$\sigma_{MPHP\_B}$	0.67 (0.032, 2.20)	0.017 (0.013, 0.022)
$\sigma_{MPHP\_U}$	0.67 (0.032, 2.20)	0.0017 (0.0014, 0.0021)
$\sigma_{OH\_U}$	0.67 (0.032, 2.20)	0.0085 (0.0069, 0.011)
$\sigma_{cx\_U}$	0.67 (0.032, 2.20)	0.00042 (0.00034, 0.00052)

**TABLE 5:** Individual-specific prior and posterior parameter distributions.

Parameter	Prior	Ind a	Ind B	Ind D	Ind F
K1_DPHP_Liver	6.82 (0.33, 22.59)	0.703 (0.021, 5.21)	5.99 (0.255, 21.0)	3.37 (0.114, 16.47)	5.18 (0.46, 17.74)
FracDOSEHep	0.202 (0.011, 0.39)	0.221 (0.202, 0.239)	0.054 (0.045, 0.066)	0.036 (0.029, 0.045)	0.103 (0.092, 0.113)
BELLYPERM	3.72 (0.24, 7.30)	0.329 (0.07, 0.84)	0.67 (0.07, 3.31)	0.57 (0.07, 3.07)	2.75 (0.49, 5.91)
GIPERM1	4.98 (0.28, 9.74)	1.79 (0.57, 9.49)	3.77 (0.95, 9.58)	2.54 (0.65, 9.49)	7.54 (3.02, 9.88)
GIPERM2	15.17 (0.72, 29.20)	0.75 (0.66, 0.97)	14.64 (2.48, 28.96)	17.13 (3.46, 29.39)	19.50 (7.15, 29.43)
Gutlag	2.51 (0.13, 4.87)	2.99 (2.89, 3.10)	1.81 (1.50, 2.13)	2.57 (2.22, 2.91)	2.16 (2.05, 2.25)
FracDoseLymph	0.075 (0.005, 0.146)	0.021 (0.017, 0.024)	0.002 (0.001, 0.005)	0.009 (0.005, 0.014)	0.010 (0.006, 0.014)
Lymphlag	3.50 (0.18, 6.81)	6.47 (6.17, 6.65)	3.88 (1.03, 5.82)	3.60 (2.99, 4.72)	2.99 (2.52, 3.69)
K1_Lymph	1.53 (0.13, 2.92)	2.58 (1.84.2.92)	1.01 (0.10, 2.86)	0.46 (0.21, 1.47)	0.69 (0.42, 1.86)
MPY	34.0 (14.54, 53.77)	30.97 (19.50, 49.10)	36.79 (21.60, 54.50)	39.05 (21.16, 58.28)	39.17 (22.82, 57.30)
MPYgu	3.92 (0.58, 7.79)	0.525 (0.22, 1.33)	1.73 (0.19, 4.52)	2.49 (0.58, 4.76)	4.66 (2.92, 6.97)
VBldC	0.05 (0.031, 0.070)	0.04 (0.03, 0.05)	0.05 (0.032, 0.070)	0.054 (0.037, 0.072)	0.048 (0.032, 0.067)
VliC	0.03 (0.011, 0.05)	0.018 (0.010, 0.028)	0.032 (0.018, 0.047)	0.033 (0.016, 0.047)	0.033 (0.018, 0.048)
VguC	0.015 (0.010, 0.020)	0.17 (0.014, 0.02)	0.015 (0.011, 0.019)	0.013 (0.010, 0.018)	0.011 (0.01, 0.014)
VkiC	0.0058 (0.0028, 0.007)	0.0059 (0.0042, 0.0078)	0.006 (0.0036, 0.0081)	0.0047 (0.003, 0.0073)	0.006 (0.004, 0.008)
QguC	0.15 (0.089, 0.21)	0.19 (0.14, 0.23)	0.16 (0.12, 0.22)	0.17 (0.12, 0.22)	0.22 (0.17, 0.24)

The second facet of model specification is the statistical error model. As described earlier, the final calibration model utilised data from four of the six individuals with data on five specific outputs formally compared within the error model. Concentrations of DPHP and MPHP (*CBlood DPHP* and *CBlood MPHP*) (mg/L) and the rates of deposition of MPHP, OH-MPHP and cx-MPHP (mg/h) into the urine (*RUrine MPHP*, *RUrine OH* and *RUrine* cx) were computed from the raw data of (Klein et al., 2018), as described earlier, and compared with equivalent predictions extracted from the PBPK model through Eqs. 1–5.

The terms ${CBloodDPHP}_{ij}$ , ${CBloodMPHP}_{ij}$ , ${RUrineMPHP}_{ij}$ , ${RUrineOH}_{ij}$ ad ${RUrinecx}_{ij}$ denote measurement i (at time $t_{i}$ ) for individual j (for j *in 1:4*) for the five respective model outputs, whereas $\mu_{DPHP\_B}{\left(\theta ,\omega_{j}\right)}_{ij}$ , $\mu_{MPHP\_B}{\left(\theta ,\omega_{j}\right)}_{ij},$ $\mu_{MPHP\_U}{\left(\theta ,\omega_{j}\right)}_{ij}$ , $\mu_{OH\_U}{\left(\theta ,\omega_{j}\right)}_{ij}$ and $\mu_{cx\_U}{\left(\theta ,\omega_{j}\right)}_{ij}$ , denote the predictions from the PBPK model corresponding to parameters ( $\theta ,\omega_{j}$ ). Normal distributions, truncated at zero were assumed for all five relationships and where $\sigma_{DPHP\_B}$ , $\sigma_{MPHP\_B}$ , $\sigma_{MPHP\_U}$ , $\sigma_{OH\_U}$ and $\sigma_{cx\_U}$ denote the respective error standard deviations

$${CBloodDPHP}_{ij}∼N\left(\mu_{DPHP\_B}{\left(\theta ,\omega_{j}\right)}_{ij},\sigma_{DPHP\_B}\right)\left[0,\infty \right]$$ (1)

$${CBloodMPHP}_{ij}∼N\left(\mu_{MPHP\_B}{\left(\theta ,\omega_{j}\right)}_{ij},\sigma_{MPHP\_B}\right)\left[0,\infty \right]$$ (2)

$${RUrineMPHP}_{ij}∼\;N\left(\mu_{MPHP\_U}{\left(\theta ,\omega_{j}\right)}_{ij},\sigma_{MPHP\_U}\right)\left[0,\infty \right]$$ (3)

$${RUrineOH}_{ij}∼\;N\left(\mu_{OH\_U}{\left(\theta ,\omega_{j}\right)}_{ij},\sigma_{OH\_U}\right)\left[0,\infty \right]$$ (4)

$${RUrinecx}_{ij}∼\;N\left(\mu_{cx\_U}{\left(\theta ,\omega_{j}\right)}_{ij},\sigma_{cx\_U}\right)\left[0,\infty \right]$$ (5)

Weakly informative, half-normal prior distributions with standard deviations of 1 were assumed for the five standard deviation parameters in Eqs. 1–5.

Inference for the model parameters was made using Markov chain Monte Carlo (MCMC) implemented in MCSim (see *Software*). Inference for model parameters in the final calibration model was made using thermodynamic integration (TI) as described in (Bois et al., 2020). A single chain of 150,000 iterations was run with every 10th retained.

### 3.4 Software

The PBPK model was written in the GNU MCSim language (version 6.1.0)^1^ and run under Windows 10 Pro. Files for running MCSim under windows, tools and instructions for installation are available from Github^2^. Scripts were written for calling the model from RStudio (RStudio Team 2016). R version 4.0.2 and the ggplot2, DiceDesign, Sensitivity and reshape2 packages were used in analysis (Dupuy et al., 2015; Pujol and Iooss 2015; R Development Core Team 2008; Wickham 2007; 2016).

## 4 Results

### 4.1 Uncertainty and sensitivity analysis

Extensive results from uncertainty and sensitivity analysis of our earlier PBPK model for DPHP are presented in McNally et al. (2021). We therefore limit our attention to the novel results from this analysis; our efforts to better fit the trends of MPHP in venous blood from the human volunteer study of Klein et al. (2018) and the change to model form so that the rate of deposition of MPHP into the urine could be simulated.

**FIGURE 5:**
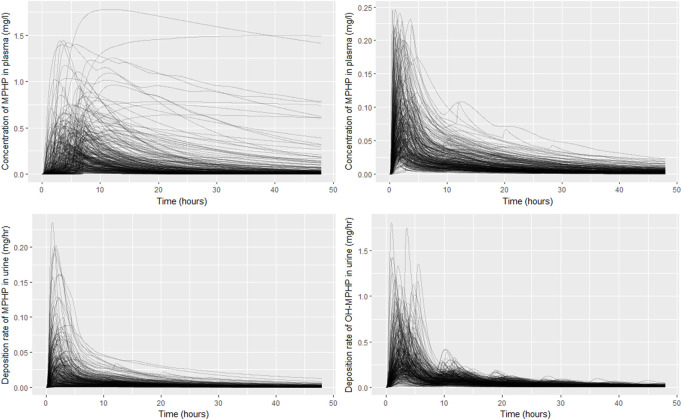
Comparisons of PBPK model predictions corresponding to the Latin Hypercube sample of 500 model runs. Panel **(A)** shows the concentration time profiles for MPHP in venous blood (mg/L) for the 500 samples; panel **(B)** shows the retained sample of 248 profiles for MPHP in venous blood (mg/L) following the rejection step; panel **(C)** shows rate of deposition of MPHP into the urine (bladder) (mg/h) for the retained sample; panel **(D)** shows rate of deposition of OH-MPHP into the urine (bladder) (mg/h) for the retained sample.

The results from uncertainty analysis are shown in Figure 5. Panel Figure 5A shows the concentration response profiles for MPHP in venous blood (mg/L) for the full 500 samples generated using a computationally efficient Latin hypercube design. The profiles indicate a wide range of behaviour for this output that is consistent with the prior distributions for model parameters. After applying the rejection criteria, specified in methods, 248 simulations were retained, and this subset of concentration response profiles is shown in Figure 5B; this analysis indicated that the model could simulate concentration-time profiles for MPHP in venous blood that were broadly consistent with the BM data of Klein et al. (2018); Figures 5C, D show predictions from the retained sample for the rates of deposition of MPHP and OH-MPHP into the urine: this analysis aims to study whether the modifications necessary to better fit the data trends in venous blood compromised the ability of the model to fit trends in urine data. These results (Figures 5C, D) showed considerable variation in the profiles from the different runs in the retained sample, with a subset of the simulations appearing to be broadly consistent with trends in BM data observed in urine data (Klein et al., 2018).

**FIGURE 6:**
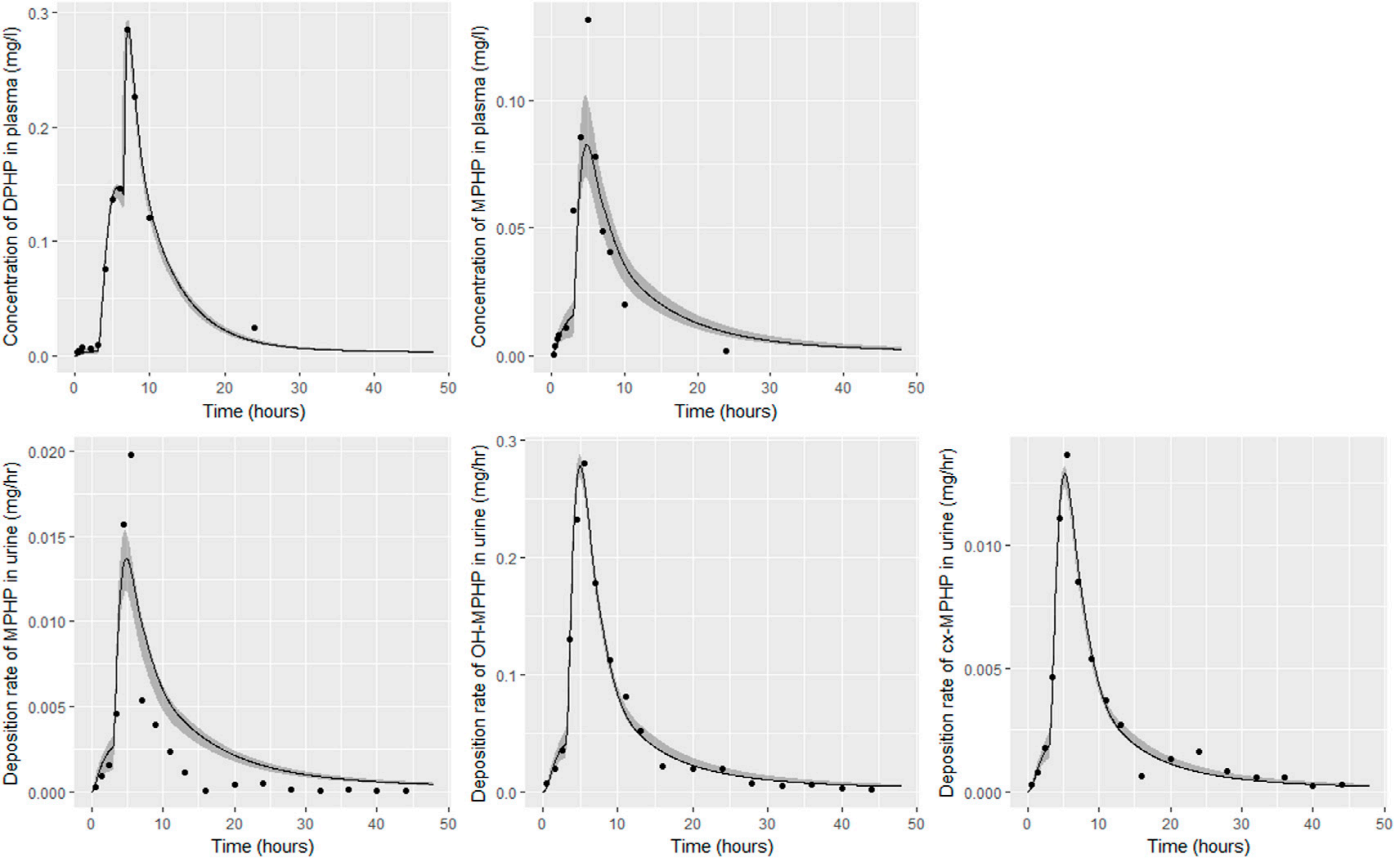
Fit of the calibrated model to data from individual **(A)**. Panel **(A)** DPHP in venous blood (mg/L); **(B)** MPHP in venous blood (mg/L); **(C)** rate of deposition of MPHP into the bladder; **(D)** rate of deposition of OH-MPHP into the bladder; and **(E)** rate of deposition of cx-MPHP into the bladder. The central estimates indicated in plots correspond to the posterior mode whereas the shaded regions represent 95% intervals for the respective curves.

**FIGURE 7:**
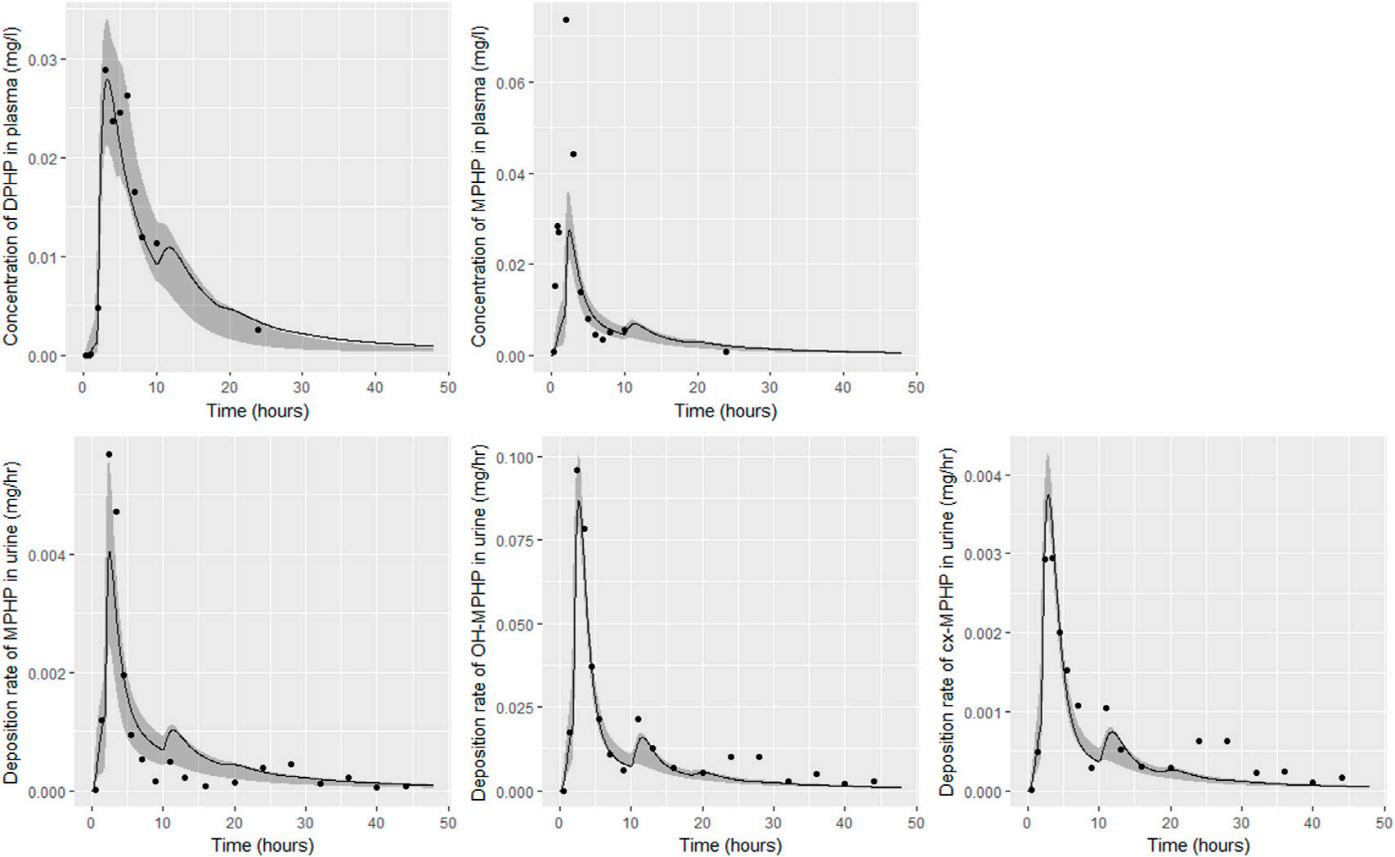
Fit of the calibrated model to data from individual **(B)**. Panel **(A)** DPHP in venous blood (mg/L); **(B)** MPHP in venous blood (mg/L); **(C)** rate of deposition of MPHP into the bladder; **(D)** rate of deposition of OH-MPHP into the bladder; and **(E)** rate of deposition of cx-MPHP into the bladder. The central estimates indicated in plots correspond to the posterior mode whereas the shaded regions represent 95% intervals for the respective curves.

**FIGURE 8:**
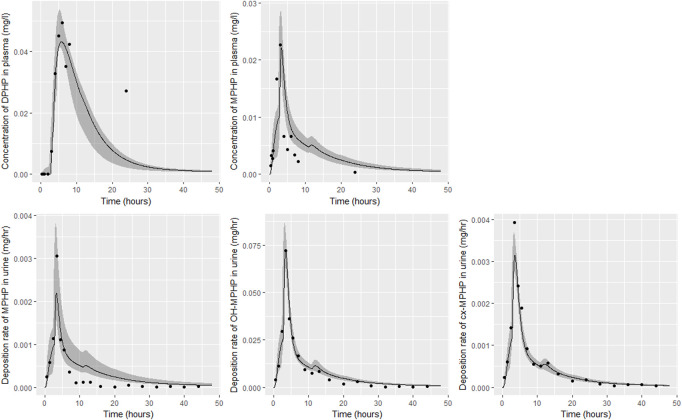
Fit of the calibrated model to data from individual **(D)**. Panel **(A)** DPHP in venous blood (mg/L); **(B)** MPHP in venous blood (mg/L); **(C)** rate of deposition of MPHP into the bladder; **(D)** rate of deposition of OH-MPHP into the bladder; and **(E)** rate of deposition of cx-MPHP into the bladder. The central estimates indicated in plots correspond to the posterior mode whereas the shaded regions represent 95% intervals for the respective curves.

**FIGURE 9:**
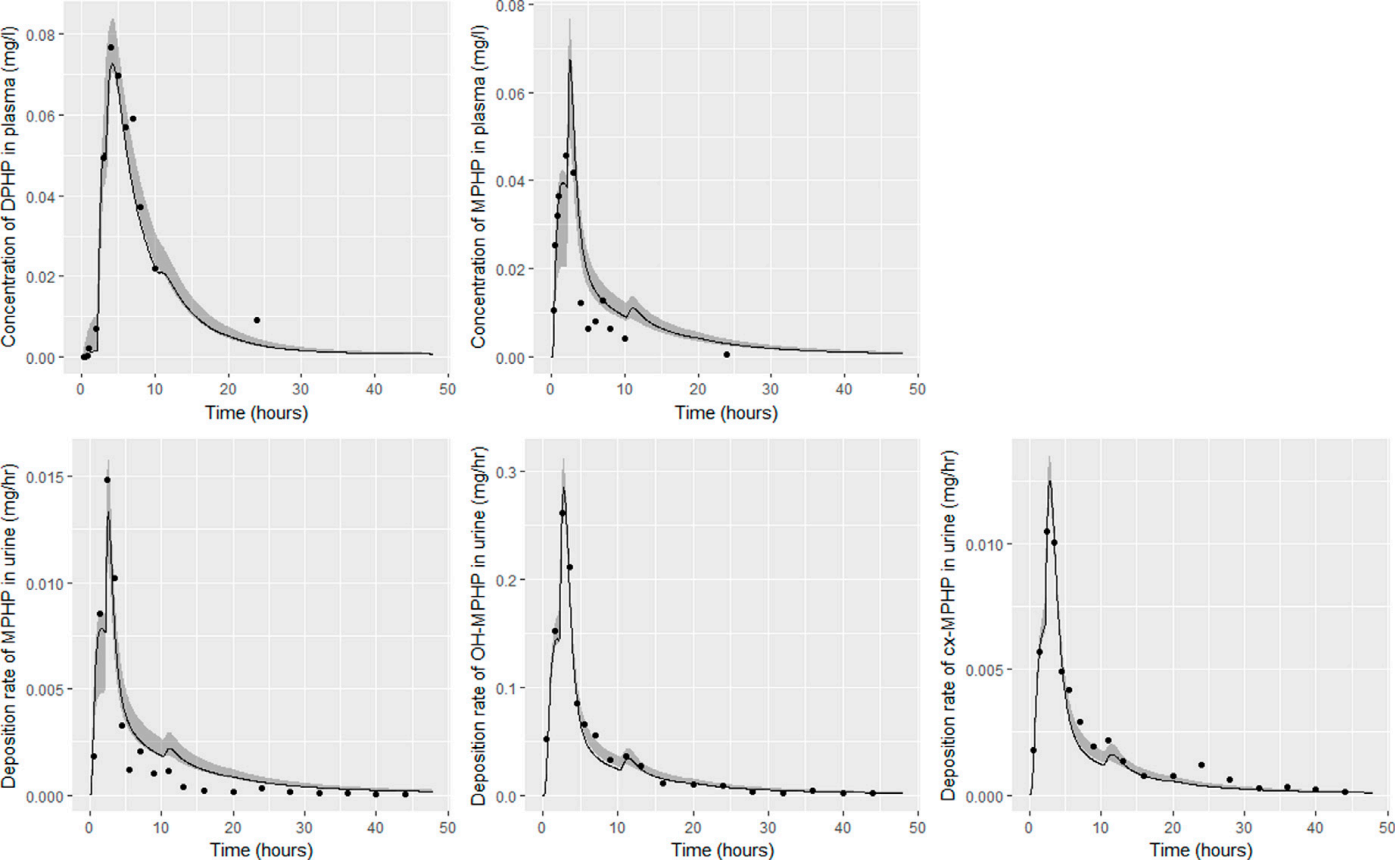
Fit of the calibrated model to data from individual **(F)**. Panel **(A)** DPHP in venous blood (mg/L); **(B)** MPHP in venous blood (mg/L); **(C)** rate of deposition of MPHP into the bladder; **(D)** rate of deposition of OH-MPHP into the bladder; and **(E)** rate of deposition of cx-MPHP into the bladder. The central estimates indicated in plots correspond to the posterior mode whereas the shaded regions represent 95% intervals for the respective curves.

Detailed results from elementary effects screening are not provided, however briefly this work aimed to explore whether additional parameters should be taken forward into calibration. The analysis was limited to concentrations of MPHP in venous blood (mg/L) at 0.5- and 3- hours and the rate of deposition of MPHP in the bladder (mg/h) also at 0.5- and 3-h following ingestion of DPHP. The proportion of MPHP unavailable for metabolism due to partial binding (escapeFracgu), the fraction of MPHP escaping from the liver due to saturation or inhibition of metabolism (escapeFracli), the mass of the kidney (as a fraction of body weight) (Vki), the blood:kidney partion coefficient for MPHP (PkiM) and the elimination rate of MPHP from kidney tissue (K1_MPHP) were identified as additional parameters to tune in model calibration. In total, the five model outputs (Figures 6–9) used in calibrations showed some sensitivity to 34 parameters (Tables 4 and 5).

### 4.2 Calibration

Summary statistics based upon the retained sample (posterior median and a 95% credible interval) for the global and local (volunteer specific) parameters are provided in Tables 4, 5 respectively. The fit of the calibrated model is shown in Figures 6–9 for individuals A, B, D and F respectively. The five panels in each figure correspond to A) DPHP in venous blood (mg/L); B) MPHP in venous blood (mg/L); C) deposition of MPHP into urine (mg/h); D) deposition of OH-MPHP in urine (mg/h); E) deposition of cx-MPHP in urine (mg/h). The central estimates indicated in plots correspond to the posterior mode parameter set, the single best fitting parameter set over the 20 measures (5 outputs for each of 4 individuals) used for calibration. The shaded regions represent pointwise 95% credible intervals for the respective curves. This interval was derived by running each retained sample drawn from the posterior through the PBPK model and storing the output from each model output from 0 to 48 h in 0.05- hour increments. Output at each time point was ordered with the 2.5th and 97.5th percentiles read off; the plotted 2.5% and 97.5% bounds are a smooth interpolation of these series of pointwise values.

The fits to BM data shown in Figures 6–9 demonstrate the PBPK model was able to simulate the diverse trends of DPHP and downstream metabolites in the blood and urine specimens from the four volunteers. A very high quality of fit to the deposition rates of OH-MPHP and cx-MPHP in urine was previously achieved in (McNally et al., 2021) and this has not been improved upon with the updated model. However, the fits to DPHP and MPHP in blood represent very significant improvements compared to those of (McNally et al., 2021). The shape of the data series on MPHP was generally captured for the four volunteers, however the model generally struggled to simulate the very rapid reduction in concentrations following the peak—the discrepancy was greatest for individual A (Figure 6), The new calibration measure considered in this work, rate of deposition of MPHP in urine (panel C of Figures 6–9), was generally well fitted, although as with MPHP in blood, the model struggled to capture the very sharp rate of decline following the peak.

The two parameters that were most critical to the improvement relative to (McNally et al., 2021) were the proportion of MPHP binding in blood within the gut and unavailable for first pass metabolism in the liver (Escape_gu), and the proportion of MPHP escaping from the liver due to saturation or inhibition of metabolism (Escape_li). In the absence of these parameters the model was unable to approximate the trends of MPHP in blood. The uniform priors U(0,1) for these parameters substantially narrowed following calibration with estimates of 0.59 (0.31, 0.83) and 0.59 (0.45, 0.75) for Escape_gu and Escape_li respectively, suggesting that a majority fraction of MPHP evades first pass metabolism in the liver, and binds with plasma, however it is subsequently rapidly removed.

## 5 Discussion

The first PBPK model for DPHP is described in McNally et al. (2021). Whilst this model described the important physiological mechanisms and provided a good fit to biological monitoring data from urine voids, the simulations of DPHP and in particular MPHP in venous blood were poorer. Given that the adverse effects that were observed with other phthalates were related to metabolism of the parent phthalate to the primary monoester (Oishi and Hiraga 1980; Foster et al., 1981; Sjöberg et al., 1986) the inability to credibly simulate MPHP, and thus interpret any potential future *in-vitro* data on DPHP toxicity, represented an important deficiency of the existing published PBPK model for DPHP.

In this work a further round of model development, based upon the published PBPK model (McNally et al., 2021), was undertaken. A few simplifications of the existing model, including the removal of EHR of MPHP, were made. However, the primary development was testing of the hypothesis that partial binding of MPHP following uptake of DPHP and metabolism in the gut might allow the model to better replicate the trends observed in BM data. However, equations for metabolism were based on clearance, parameterised through a half-life. A relatively short half-life was estimated for MPHP from *in-vitro* experimental data. A problem with this specification is that the domain of applicability of the model is limited by the metabolic clearance term which is linear at relatively low tissue concentrations but ceases to be relevant at higher concentrations of chemical since the equation does not account for saturation or inhibition of metabolism. The BM data suggested that a partial limitation to the metabolism of MPHP, for which we speculate may be saturation or partial inhibition. A very sharp spike was observed in blood concentrations of volunteers before rapid removal. This feature of the data could not be explained by McNally et al. (2021). A simple approach was coded in our model to specify a fraction of MPHP that was unavailable for first pass metabolism in the liver. Whilst we recognise that metabolism described through Michaelis-Menten kinetics is a better approach, we lacked the high-quality *in-vitro* derived metabolic rate constants that could be scaled up to the whole liver. Consequently, whilst the constant estimated in our model specification is suitable for the oral dose ingested by study volunteers, there is no natural mechanism in the model to allow for dosage corrections. At the much lower dosage associated with environmental exposures to plasticizers, complete first-pass metabolism of MPHP is likely—the MPHP spike observed in the BM study should not manifest. Results from model calibration suggest a satisfactory fit to BM data of Klein et al. (2018) was achieved with the appearance of MPHP in blood, and its subsequent removal in urine.

The final model is therefore considered suitable for contextualising any human *in-vitro* data of DPHP or MPHP that may be available in the future. There are a small number of simplifications in the model. The removal of MPHP from the kidney and OH-MPHP and cx-MPHP from blood is simplified by using first-order rate constants; ideally the model would attempt to encode biological mechanisms. Furthermore, OH-MPHP and cx-MPHP are treated as fractions of metabolised MPHP and there is no detailed modelling of the kinetics of these two metabolites, nor did we use BM data from a third metabolite, oxo-MPHP which is a downstream metabolite of OH-MPHP. Further development of our model is possible, however direct information on kidney clearance mechanisms and metabolism data would be required to effect further changes. The time and effort of further *in-vitro* work and PBPK model refinement would only be justified if specific concerns were raised about OH-MPHP, cx-MPHP or oxo-MPHP that necessitated tissue concentrations for these chemicals.

We finish this work with a discussion of some features of the data and some comments on the calculation of a reference intake dose (RfD) for DPHP (of 0.1 mg/kg-day), which was derived from the human equivalent 10% benchmark dose lower bound of the confidence level (BMDL_10_) of 10 mg/kg-day for thyroid hypertrophy/hyperplasia in male F1 adults from a two-generation study (Bhat et al., 2014).

Bhat et al. (2014) observed that ‘*DPHP behaves similarly to other intermediate or high molecular weight phthalate esters such as DEHP, di-iso-nonylphthalate (DINP) and di-n-octyl-phthalate (DNOP), which are readily hydrolyzed in the gastrointestinal tract and absorbed as their corresponding monoesters in rodents and subsequently metabolized to more hydrophilic oxidative metabolites excreted mainly in the urine. Only between 1% and 7% of the dose is excreted as the simple monoester for long-chain phthalates such as DPHP, DEHP, DINP, and di-iso-decylphthalate (DIDP)*’ Our model suggests that some aspects of the behaviour in rodents carry over to human populations—specifically that absorbed DPHP is mainly eliminated in the urine as more hydrophilic oxidative metabolites—however the absorption of DPHP in humans is more complex. There is certainly evidence from urine data on MPHP, cx-MPHP and OH-MPHP and blood data on MPHP that a proportion of DPHP is hydrolyzed in the gut and absorbed as MPHP. However, in the study reported in (Klein et al., 2018) DPHP was not ‘readily absorbed’, with only a small fraction of between 4.6% (3.4, 6.0) and 24.2% (21.7, 26.3) of DPHP absorbed by the study participants. In an earlier bio-monitoring study reported in Leng et al. (2014) a mean of 24% of DPHP, over twice the average from the Klein et al. (2018) study, was eliminated as second order metabolites in urine voids over a 48 h period. This study also found very large variability in the fraction absorbed over the study volunteers. Both the food intake of volunteers and the study protocol, specifically in how DPHP was administered, appear to strongly influence uptake from the gut, however the relatively small fractions of DPHP absorbed suggest it is not ‘readily absorbed’ from the gut. The combination of the form of DPHP administration and small fraction absorbed can lead to the requirement for the use of individual parameter values sampled from the extreme ends of the physiologically feasible ranges of the posterior distributions, as was the case for volunteer A. More importantly the BM data from OH-MPHP and cx-MPHP in urine, suggest a two-phased uptake with a marked acceleration in the production of these metabolites (Figures 6—9). Coupled with the evidence for EHR from OH-MPHP and cx-MPHP in urine, these findings suggest uptake of DPHP in a lower section of the small intestine with metabolism (and EHR) in the liver occur in human subjects. Our model suggests that the majority of metabolism of DPHP occurs in the liver as opposed to the gut. Finally, evidence from DPHP in blood, in particular volunteers A and F (Figures 6, 9) suggests a small fraction of DPHP is taken up *via* the lacteals in the gut and into the lymphatic system before draining into blood at the thoracic duct, thus bypassing first pass metabolism and rapidly binding in blood. Due to the high binding and small fraction of DPHP entering *via* this route (approximately 1/8 of that entering *via* the hepatic route) this artefact was only visible in the series from DPHP in blood: this artefact was previously explored in McNally et al. (2021).

The appearance of the peak concentration of the primary metabolite before the parent chemical is counter-intuitive behaviour and would not have been identified using the urinary excretion data only. Modification of the model to include biological mechanisms to simulate the pharmacokinetics of a chemical and/or metabolites observed in one medium and not another could imply a significant and important lack of knowledge. A PBPK model developed for a “data rich” source chemical would have a structure developed, calibrated, and validated against multiple data streams i.e., blood, breath, and urine. The formulation of a set of rules could also help in defining an initial model structure, for example, the inclusion of lymphatic uptake in the gut for highly lipophilic chemicals like the phthalates. These observations have important implications for the “read across” approach proposed as part of the development of New Approach Methods for the replacement of animals in chemical safety assessments. This is where the endpoint of a target chemical is predicted by using data for the same endpoint from another more “data rich” source chemical. Validation of a model parameterized entirely with *in vitro* and *in silico* derived parameters and calibrated against several data streams would constitute a data rich source chemical and afford more confidence for future evaluations of other similar chemicals using the read-across approach.

We finish with brief comments on the exclusion of data from volunteers C and E; these data were excluded due to the suspicion that a second significant uptake event, associated with food intake, occurred for these two individuals, with the PBPK model unable to capture the complex trends in BM data. There was evidence of secondary uptake events for other volunteers, notably at the 24-h period evidenced by a high DPHP concentration in blood at this time point and a rise in the deposition of OH-MPHP and cx-MPHP in urine specimens at a similar time-point. Due to the relatively low concentrations and the time point associated with such events in the study, this is assessed as having a negligible impact on results from calibration—it was simply a data artefact that could not be fitted without further modification of the model. However, secondary uptake events have been investigated in further exploratory analysis—we show how a small additional uptake from the gut at 24 h following ingestion of DPHP might appear in urine using the model calibration output for individual C in Supplementary Figure S1. Although we have to acknowledge there is weak direct evidence for secondary uptake events, the ability of the model to successfully fit these unusual quirks of the BM data does provide indirect evidence that DPHP can be taken up from lower sections of the intestine following consumption of food for an extended period following ingestion.

## Data Availability

The original contributions presented in the study are included in the article/Supplementary Material, further inquiries can be directed to the corresponding author.

## Footnotes

1
https://www.gnu.org/software/mcsim/(16/08/2022).

2
https://github.com/GMPtk/MCSimViaRtools.
