# Supplementary material for: Refinement and calibration of a human PBPK model for the plasticiser, Di-(2-propylheptyl) phthalate (DPHP) using in silico, in vitro and human biomonitoring data
Source: Front Pharmacol. 2023 Feb 2;14:1111433. doi: 10.3389/fphar.2023.1111433 (PMC9971821; doi:10.3389/fphar.2023.1111433)
Supplement: Supplementary file 1 [file DataSheet1.docx]

# Supplementary Materials

Refinement and calibration of a human PBPK model for the plasticiser, Di-(2-propylheptyl) phthalate (DPHP) using in silico, in vitro and human biomonitoring data

Kevin McNally and George Loizou

## Materials and Methods

#### Anatomical

Baseline estimates of organ and tissue masses and regional blood flows were taken from Brown et al. (1997) and ICRP (2002).

#### Physicochemical

The logarithms of the octanol–water partition coefficient, log P_ow_ for DPHP and MPHP were estimated using the ACDLogP algorithm (Mannhold *et al.*, 2009) implemented in the ACD/ChemSketch 2014 software (Table 1). The tissue:blood PCs and unbound fractions in plasma were calculated from log P_ow._ The log P_ow_ values were input into two tissue-composition-based algorithms for the calculation of tissue:blood PCs. The method of Poulin and Haddad (2012), which was developed for the prediction of the tissue distribution of highly lipophilic compounds, defined as chemicals with a Log P_ow_ > 5.8, was used for DPHP (Table 1). The method of Schmitt (2008), which was developed to predict the tissue distribution of chemicals with log P_ow_ < 5.17, was used to predict the PCs of the monoester, MPHP (Table 1). The algorithm of Poulin and Haddad (2012) was implemented as a Microsoft^®^ Excel Add-in whereas a modified version of the algorithm of Schmitt (2008) was available within the httk: R Package for High-Throughput Toxicokinetics (Pearce *et al.*, 2017). Where the tissue-composition-based algorithms did not provide a tissue:blood partition coefficient for a particular compartment, the value from a surrogate organ or tissue was assumed. These are presented in italicised text with the surrogate organ or tissue in brackets Table 1.

The fraction unbound (*fu*) was calculated from *log ((1-fu)/fu)* and based on information from Table 1 of (Lobell and Sivarajah, 2003) (Table 1), corresponding to chemicals with a predominantly uncharged state at pH 7.4. Baseline values of *fu* were calculated for both DPHP and MPHP.

|  | $fu=\frac{1}{{10}^{0.4485logP_{ow}-0.4782}+1}$ | (1) |
| --- | --- | --- |

#### Metabolism

Metabolism of MPHP in the liver was modelled through using clearance. The in-vitro experimental set-up for the calculation of in-vitro clearance ${CL}_{in vitro}$ (ml min^-1^ mg^-1^ microsomal protein) in human hepatic microsomes (Obach *et al.*, 1997) is described in McNally et al. (2021). The key quantity determined from in-vitro experiments was the half-life (T_1/2)_ of MPHP in the system. The Intrinsic hepatic clearance was calculated using (2).

|  | ${CL}_{int\_H}={CL}_{in vitro}\times MPY\times Vli\times60$ | (2) |
| --- | --- | --- |

where, *MPY* is the microsomal protein yield per g liver tissue (mg g^-1^), *Vli* is mass of the liver (g) and the 60 converts from minutes to hours. Finally, whole liver plasma clearance *CL_H_* (L h^-1^) was calculated assuming the well-stirred model of hepatic clearance (Yang *et al.*, 2007), taking into account the unbound fraction in plasma, *fu* and the red blood cells to plasma ratio, C_RBC_/C_P_,

|  | ${CL}_{H}=Q_{H}\cdot fu\cdot\frac{{CL}_{int\_H}}{Q_{H}+fu\cdot{{CL}_{int\_H}}/{({C_{RBC}}/{C_{p}})}}$ | (3) |
| --- | --- | --- |

Where, *Q_H_* (L h^-1^) is the blood flow to the liver as a proportion of cardiac output.

Metabolism of DPHP was described in the liver using similar equations to (2)-(3) and differing only in the fraction unbound (eq.2) and the half-life. Following technical difficulties in the in-vitro experiments due to the extremely high lipophilicity of DPHP (McNally et al., 2021), the half-life of DPHP was initially estimated as being equal to that of MPHP.

Metabolism of DPHP in the gut was also described using similar equations (4)-(5), where *Q_Gu_* (L h^-1^) is the blood flow to the gut as a proportion of cardiac output, *MPYgu* is the microsomal protein yield per g gut tissue (mg g^-1^), and *Vgu* is mass of the gut (g). A half-life was initially estimated as being equal to that of MPHP.

| ${CL}_{Gu}=Q_{Gu}\cdot fu\cdot\frac{{CL}_{int\_Gu}}{Q_{Gu}+fu\cdot{{CL}_{int\_Gu}}/{({C_{RBC}}/{C_{p}})}}$ | (4) |
| --- | --- |
| ${CL}_{int\_Gu}={CL}_{in vitro}\times MPYgu\times VGu\times60$ | (5) |
|  |  |

#### Constants

The proportion of MPHP metabolised to cx- and OH-MPHP, represented by FracMetab (FracMetabcx to cx- MPHP and FracMetabOH to OH-MPHP) (Table 2) for each volunteer was estimated by expressing all the biological monitoring (BM) data (MPHP, OH-MPHP, cx-MPHP, oxo-MPHP) in moles and dividing the amount of cx- and OH-MPHP each by the sum total of all metabolites (Table 2).

A single term for metabolism of MPHP was coded in the PBPK model (2) with the rates of removal of two direct metabolites, (OH-MPHP and cx-MPHP), from plasma assumed to be proportional to the rate of metabolism of MPHP. A urinary elimination constant was coded for OH-MPHP and cx-MPHP. Baseline values were fitted based upon the model values for surrogate metabolites of the plasticizer DINCH (McNally et al, 2019).

Baseline values for parameters for which there was no prior knowledge such as *FracDOSEHep, FracDOSELymph* and the various delay terms and uptake and elimination rates were determined during the model development and testing process to provide a reasonable (but not optimised) fit to BM data.

Baseline (default) values are given in Table 3.


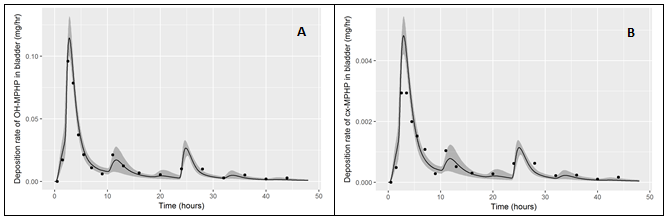


Figure S1 Secondary gut uptake event observed at 24 hours following ingestion of DPHP in two of the volunteers (C and E).

## Model Code

# MCSim 6.1.0 model of Di(2-propyl heptyl) phthalate

# Compiled on 27/02/20.

# Translated version of an R model.

# New inclusion is a simplified two compartment model of the gut for absorption of DPHP and MPHP

States =

{

Afa,

Agu1,

Agu2,

AMgu,

Ast,

Arpd,

Aspd,

Ali,

Aki,

AMli,

ABile,

Alymph,

ARBC_DPHP,

Aplasm_DPHP,

ABellyH,

AGiTractH,

ABowel,

ABellylymph,

AGiTractlymph,

AfaM,

AstM,

AguM,

AMliM,

AliM,

AkiM,

ABileM,

ABowelM,

AspdM,

ArpdM,

Aplasm_MPHP,

ARBC_MPHP,

AMMPHPB_MOH,

AMMPHPB_cx,

AMMPHPU_MOH,

AMMPHPU_cx,

VBladder,

Gutswitch,

Lymphswitch,

Bowelswitch,

DOSESTEP

};

Outputs =

{

Cli,

Cki,

Cfa,

Cgu1,

Cgu2,

Cst,

Clymph,

Cspd,

Crpd,

mass,

Uptake,

reloral,

CVfa,

CVgu1,

CVgu2,

CVst,

CVspd,

CVli,

CVki,

CVrpd,

CV,

CVnmol,

CA_DPHP,

CAT_DPHP,

CARBC_DPHP,

CV_total_nmol,

CVM,

CliM,

CkiM,

CVliM,

CVkiM,

CguM,

CVguM,

CstM,

CVstM,

CfaM,

CVfaM,

CspdM,

CVspdM,

CrpdM,

CVrpdM,

CA_MPHP,

CRBC_MPHP,

CRBC_DPHP,

Curine_MPHP,

Curine_MOH,

Curine_cx,

ODOSEliver,

ODOSElymph,

ODOSEbowel,

ClintDPHP,

ClintDPHPgu,

ClintMPHP,

Ali_lag,

AliM_lag,

Blood_DPHP,

Blood_MPHP,

Blood_cx,

Blood_OH,

Urine_cx,

Urine_OH,

Urine_MPHP

};

Inputs =

{

events_Gutswitch,

events_Lymphswitch,

events_Bowelswitch,

events_DOSESTEP,

events_VBladder,

events_AMMPHPU_MOH,

events_AMMPHPU_cx

};

# Parameters

# ==========

#

BW = 89; # body mass (kg)

MWDPHP = 446.67; # DPHP molecular mass (g/mol)

MWMPHP = 306.41; # MPHP molecular mass (g/mol)

MWMPHPOH = 322.39; # OH-MPHP molecular mass (g/mol)

MWMPHPcx = 336.37; # cx-MPHP molecular mass (g/mol)

CAE = 0.75; # cardiac allometric exponent

QCC = 11.22; # cardiac allometric constant (L/h/kg^CAE)

VT = 0.95; # proportion of vascularised tissue

VfaC = 0.195; # fractional volume

VguC = 0.067; # fractional volume

VstC = 0.0158; # fractional volume

VspdC = 0.4714; # fractional volume poorly perfused

VrpdC = 0.0305; # fractional volume richly perfused

VliC = 0.0203; # fractional volume

VkiC = 0.0025; # fractional volume kidney

VlymphC = 0.0036; # lymph system fractional volume

VBldC = 0.05; # blood fractional volume

QhepartC = 0.06; # hepatic artery fractional blood flow

QguC = 0.17; # fractional blood flow

QstC = 0.01; # fractional blood flow

QspdC = 0.27; # overall fractional blood flow to slowly perfused tissue

QrpdC = 0.22; # overall fractional blood flow to rapidly perfused tissue

QkiC = 0.2; # fractional blood flow to kidney

QfaC = 0.05; # fractional blood flow

FracDOSELymph = 0.05; # Fraction of dose taken into lymph

FracDOSEHep = 0.1; # Fraction of dose taken into hepatic

FracMetabMOH = 0.3; # Fraction of CYP-mediated metabolism MPHP -> MPHP (Table 2 Koch et al (2013) Arch Tox 87) 0.25

FracMetabcx = 0.05; # Fraction of CYP-mediated metabolism MPHP -> MPHP (Table 2 Koch et al (2013) Arch Tox 87) 0.25

FB_DPHP = 0.9975; # Fraction of DPHP bound to plasma proteins 0.9998752

FB_MPHP = 0.9854; # Fraction of MPHP bound to plasma proteins 0.9854

PORALDOSE = 0.7; # oral dose [mg/kg]

DRINKTIME = 0.05; # Drink time [h]

BELLYPERM = 0.685; # [/h]

GIPERM1 = 5.1; # [/h]

GIPERM2 = 5.1; # [/h]

BELLYPERMlymph = 0.685; # [/h]

GIPERMlymph = 5.1; # [/h]

KEMAX = 10.2; # [Maximum emptying rate /h]

KEMIN = 0.005; # [Minimum emptying rate /h]

KA_MPHP = 0.3; # 1st-order oral uptake rate of MPHP (1/hr)

Lymphswitch = 1;

Gutswitch = 1;

Bowelswitch = 1;

MPY = 34; # microsomal protein yield [mg microsomal protein/g liver]

MPYgu = 3.9; # microsomal protein yield [mg microsomal protein/g gut]

Incub_vol = 1; # Volume of incubation (ml)

Microsome_prot = 0.5; # microsomal protein amount (mg)

DPHP_half_life = 3; # DPHP -> MPHP half-life (minutes)

DPHP_GUT_half_life = 60; # DPHP -> MPHP GUT half-life (minutes)

MPHP_half_life = 8.0486; # MPHP -> OH-MPHP and cx-MPHP half-life (minutes)

RUrine = 0.1; # Rate of Urine Production [l/h]

Creat = 1.217; # Urinary creatinine concentration [g/L] or 0.01192 [mol/L]

K1_MOH = 0.1; # First-order elimination rate from blood [/h]

K1_cx = 0.1; # First-order elimination rate from blood [/h]

K1_MPHP = 0; # First-order elimination rate from plasma [/h]

K1_DPHP_GUT = 0.1; # First-order elimination rate of DPHP from gut into bowel [/h]

K1_DPHP_LIVER = 10; # First-order elimination rate of MPHP from liver into bile [/h]

K1_MPHP_GUT = 0.1; # First-order elimination rate of MPHP from gut into bowel [/h]

K1_MPHP_LIVER = 1; # First-order elimination rate of MPHP from liver into bile [/h]

K1Lymph = 0.2; # First-order elimination rate from Lyph into blood [/h]

Lymphlag = 3.01; # Lag between uptake into Lymph and emptying into blood [h]

Gutlag = 3.01; # Lag between uptake into GItract and emptying into gut [h]

Pbab = 3.01; # DPHP Red blood cells:plasma partition coefficient

Pfab = 63.38; # DPHP fat tissue:blood partition coefficient

Pgub = 7.4; # DPHP GI tract tissue:blood partition coefficient

Pstb = 7.4; # DPHP stomach tissue:blood partition coefficient

Prpdb = 3.7; # DPHP Richly tissue:blood partition coefficient

Pkib = 3.7; # DPHP kidney tissue:blood partition coefficient

Pspdb = 3.29; # DPHP Slowly perfused tissue:blood partition coefficient

Plib = 5.89; # DPHP liver tissue:blood partition coefficient

Prbcb = 30; # DPHP liver tissue:blood partition coefficient

PbaM = 6.67; # MPHP Red blood cells:plasma partition coefficient

PspdM = 7.51; # MPHP Slowly perfused tissue:blood partition coefficient

PliM = 54.8; # MPHP tissue:blood partition coefficient

PrpdM = 12.20; # MPHP Richly tissue:blood partition coefficient

PkiM = 12.20; # MPHP kidney tissue:blood partition coefficient

PfaM = 29.10; # MPHP Fat tissue:blood partition coefficient

PstM = 25.2; # MPHP Stomach tissue:blood partition coefficient

PguM = 25.2; # MPHP GI Tract tissue:blood partition coefficient

PrbcM = 30; # DPHP liver tissue:blood partition coefficient

Vfa = 0;

Vgu = 0;

Vst = 0;

Vspd = 0;

Vrpd = 0;

Vli = 0;

Vki = 0;

Vlymph = 0;

Qfa = 0;

Qgu = 0;

Qst = 0;

Qrpd = 0;

Qki = 0;

Qspd = 0;

Qli = 0;

QCMC = 0;

ODOSEliver = 0;

ODOSElymph = 0;

ODOSEbowel = 0;

Uptake = 0;

Vplas = 0;

VRB = 0;

Qhepart = 0;

CA_DPHP = 0;

CVnmol = 0;

CVM = 0;

escapeFrac = 0.05;

# SD terms for MCMC

# ==========

#

Sigma1 = 0.1;

Sigma2 = 0.1;

Sigma3 = 0.1;

Sigma4 = 0.1;

Sigma5 = 0.1;

Sigma6 = 0.1;

Initialize

{

BWc = pow(BW, CAE); # cardiac scaling output factor (kg)

VplasC = 0.55 * VBldC; # plasma fractional volume

HEME = 1 - (VplasC / VBldC); # Volume of Haeme

VRBC = HEME * VBldC; # Volume of red blood cells

## Gelman reparameterisations

Qcci = QrpdC + QspdC + QhepartC + QfaC + QstC + QguC + QkiC;

Qrpdci = QrpdC / Qcci;

Qspdci = QspdC / Qcci;

Qhepartci = QhepartC / Qcci;

Qkici = QkiC/Qcci;

Qfaci = QfaC / Qcci;

Qstci = QstC / Qcci;

Qguci = QguC / Qcci;

Vti =

(1 - VT) + VrpdC + VspdC + VliC + VkiC +

VfaC + VstC + VguC + VplasC +

VRBC + VlymphC;

Vguci = VguC / Vti;

Vstci = VstC / Vti;

Vfaci = VfaC / Vti;

Vlici = VliC / Vti;

Vkici = VkiC / Vti;

Vspdci = VspdC / Vti;

Vrpdci = VrpdC / Vti;

Vbldci = VBldC / Vti;

Vplasci = VplasC / Vti;

VRBCci = VRBC / Vti;

Vlymphci = VlymphC / Vti;

# Volumes scaled to actual volumes

Vfa = Vfaci * BW; # scaled fractional volume

Vgu = Vguci * BW; # scaled fractional volume

Vst = Vstci * BW; # scaled fractional volume

Vspd = Vspdci * BW; # scaled fractional volume

Vli = Vlici * BW; # scaled fractional volume

Vki = Vkici * BW; # scaled fractional volume

Vrpd = Vrpdci * BW; # scaled fractional volume

Vlymph = Vlymphci * BW; # scaled fractional volume

VRB = VRBCci * BW; # scaled red blood cell fractional volume

#Vplasci = 0.55 * VBldC; # plasma fractional volume

Vplas = Vplasci * BW; # plasma fractional volume

# Calculate actual blood flows from total flow and percent flows

QC = QCC * BWc; # cardiac output (L/h)

Qfa = Qfaci * QC; # scaled fractional blood flow

Qgu = Qguci * QC; # scaled fractional blood flow

Qst = Qstci * QC; # scaled fractional blood flow

Qki = Qkici * QC; # scaled fractional blood flow

Qrpd = Qrpdci * QC; # scaled fractional blood flow

Qspd = Qspdci * QC; # scaled fractional blood flow

Qhepart = Qhepartci * QC; # scaled hepatic artery fractional blood flow

Qli = Qhepart + Qst + Qgu; # scaled fractional blood flow

QCMC = Qhepart + Qgu + Qst + Qfa + Qrpd + Qspd + Qki;

} # End of model initialization

Dynamics

{

tau = 8; # the required delay

Ali_lag = CalcDelay(Ali, tau);

AliM_lag = CalcDelay(AliM, tau);

ORALDOSE = PORALDOSE * BW; # scaled oral dose (mg/day)

DOSEFLOW = ORALDOSE / DRINKTIME; # zero order uptake rate constant

ODOSE = DOSEFLOW * DOSESTEP; # amount absorbed (mg)

ODOSEliver = ODOSE * FracDOSEHep;

ODOSElymph = ODOSE * FracDOSELymph;

ODOSEbowel = ODOSE * (1 - FracDOSEHep - FracDOSELymph);

ClintDPHP = (0.693 / DPHP_half_life) * (Incub_vol / Microsome_prot) * MPY * Vli * 60; # Clearance (L/h whole liver)

ClintDPHPgu = (0.693 / DPHP_GUT_half_life) * (Incub_vol / Microsome_prot) * MPYgu * Vgu * 60; # Clearance (L/h gut)

ClintMPHP = (0.693 / MPHP_half_life) * (Incub_vol / Microsome_prot) * MPY * Vli * 60; # Clearance (L/h whole liver)

#********************************************************************************************!

# DPHP Concentrations in Compartments

#********************************************************************************************!

# cellular concentrations (mg/L)

Cfa = Afa / Vfa;

Cgu1 = Agu1 / Vgu;

Cgu2 = Agu2 / Vgu;

Cst = Ast / Vst;

Cspd = Aspd / Vspd;

Crpd = Arpd / Vrpd;

Cki = Aki / Vki;

Cli = Ali / Vli;

Clymph = Alymph / Vlymph;

# venous organ concentration (mg/L)

CVfa = Cfa / Pfab;

CVgu1 = Cgu1 / Pgub;

CVgu2 = Cgu2 / Pgub;

CVst = Cst / Pstb;

CVspd = Cspd / Pspdb;

CVli = Cli / Plib;

CVki = Cki / Pkib;

CVrpd = Crpd / Prpdb;

GPER = KEMAX / (1 + KEMIN * Cst);

# venous concentration (mg/L)

CV =

((CVfa * Qfa) +

(CVrpd * Qrpd) +

(CVspd * Qspd) +

(CVki * Qki) +

(CVli * Qli)) / QCMC;

# DPHP Venous concentration (nmoles/L)

CVnmol = (CV / MWDPHP) * 1000000;

# Fraction unbound

Aplasmub_DPHP = Aplasm_DPHP * (1 - FB_DPHP);

# mass in system (kg)

mass =

ARBC_DPHP + Aplasm_DPHP + AMli + Ali + ABile + AMgu + ABellyH + AGiTractH +

Ast + Agu1 + Agu2 + ABowel + ABellylymph + AGiTractlymph + Alymph + Afa + Arpd +

Aspd + Aki;

Uptake = ODOSEliver + ODOSElymph + ODOSEbowel;

# mass balance

reloral = ((t>0) ? mass / (ORALDOSE + 1e-10) : 1);

CA_DPHP = Aplasmub_DPHP /Vplas; # Arterial unbound concentration (nmol/L)

CRBC_DPHP = ARBC_DPHP/VRB; # Concentration in red blood cells (nmol/L)

#********************************************************************************************!

# MPHP Concentrations in Compartments

#********************************************************************************************!

# cellular concentrations (mg/L)

CguM = AguM / Vgu;

CstM = AstM / Vst;

CfaM = AfaM / Vfa;

CliM = AliM / Vli;

CkiM = AkiM / Vki;

CspdM = AspdM / Vspd;

CrpdM = ArpdM / Vrpd;

# venous organ concentration (mg/L)

CVguM = CguM / PguM;

CVstM = CstM / PstM;

CVfaM = CfaM / PfaM;

CVliM = CliM / PliM;

CVkiM = CkiM / PkiM;

CVspdM = CspdM / PspdM;

CVrpdM = CrpdM / PrpdM;

CVM =

((CVliM * Qli) +

(CVfaM * Qfa) +

(CVspdM * Qspd) +

(CVkiM * Qki) +

(CVrpdM * Qrpd)) / QCMC;

#unbound model

Aplasmub_MPHP = Aplasm_MPHP * (1 - FB_MPHP);

CA_MPHP = Aplasmub_MPHP / Vplas;

CRBC_MPHP = ARBC_MPHP / VRB;

#********************************************************************************************!

# DPHP Differential Equations

#********************************************************************************************!

dt (Gutswitch) = 0;

dt (Lymphswitch) = 0;

dt (Bowelswitch) = 0;

dt (DOSESTEP) = 0;

dt (VBladder) = RUrine;

dt (ARBC_DPHP) = (CA_DPHP - CRBC_DPHP / Prbcb); # Amount in red blood cells

#dt (ARBC_DPHP) = 0;

dt (Aplasm_DPHP) = # Amount in plasma (mg)

QCMC *

(CV - CA_DPHP) -

dt (ARBC_DPHP) +

Lymphswitch * Alymph * K1Lymph;

dt (AMli) = ((Qli * ClintDPHP) / (Qli + ClintDPHP / Pbab)) * CVli; # Amount of hepatic metabolism (mg/h/kg)

dt (Ali) = # Amount in liver (mg)

(Qhepart * CA_DPHP) +

(Qst * CVst) +

(Qgu * CVgu2) -

(Qli * CVli) -

dt (AMli) -

(K1_DPHP_LIVER * Ali);

dt (ABile) = K1_DPHP_LIVER * (Ali - Ali_lag); # Amount in Bile (mg)

dt (AMgu) = ((Qgu * ClintDPHPgu) / (Qgu + ClintDPHPgu / Pbab)) * CVgu1; # Amount of gut metabolism (mg/h/kg)

dt (ABellyH) = (ODOSEliver) - (GPER * ABellyH) - (BELLYPERM * ABellyH); # Amount of in stomach compartment (mg/h/kg)

dt (AGiTractH) = (GPER * ABellyH) - (GIPERM1 * AGiTractH); # Amount of in GI Tract compartment (mg/h/kg)

dt (Ast) = Qst * (CA_DPHP - CVst) + BELLYPERM * ABellyH; # Amount of in STOMACH compartment (mg/h/kg)

dt (Agu1) =

(GIPERM1 * AGiTractH) -

dt (AMgu) -

(Gutswitch * GIPERM2 * Agu1)

;

dt (Agu2) =

Qgu * (CA_DPHP - CVgu2) +

(Gutswitch * GIPERM2 * Agu1) -

#dt (AMgu) -

(Bowelswitch * K1_DPHP_GUT * Agu2) +

(K1_DPHP_LIVER * Ali_lag);

dt (ABowel) = ODOSEbowel + (Bowelswitch * K1_DPHP_GUT * Agu2); # Elimination rate from gut into faeces (mg)

dt (ABellylymph) = # DPHP rate of uptake in lymph compartment (mg/h/kg)

(ODOSElymph) -

(GPER * ABellylymph) -

(BELLYPERMlymph * ABellylymph);

dt (AGiTractlymph) = (GPER * ABellylymph) - (GIPERMlymph * AGiTractlymph); # DPHP rate of uptake in lymph compartment (mg/h/kg)

dt (Alymph) = # Amount in lymph

(BELLYPERMlymph * ABellylymph) +

(GIPERMlymph * AGiTractlymph) -

Lymphswitch * Alymph * K1Lymph;

dt (Afa) = Qfa * (CA_DPHP - CVfa); # cellular compartment derivative (mg/h/kg)

dt (Arpd) = Qrpd * (CA_DPHP - CVrpd); # cellular compartment derivative (mg/h/kg)

dt (Aspd) = Qspd * (CA_DPHP - CVspd); # cellular compartment derivative (mg/h/kg)

dt (Aki) = Qki * (CA_DPHP - CVki); # cellular compartment derivative (mg/h/kg)

#********************************************************************************************!

# MPHP Differential Equations

#********************************************************************************************!

dt (AMliM) = ((Qli * ClintMPHP) / (Qli + ClintMPHP / PbaM)) * CVliM;

dt (AliM) =

(Qhepart * CA_MPHP) +

(Qst * CVstM) +

(Qgu * CVguM) -

(Qli * CVliM) +

dt (AMli) -

dt (AMliM) -

(K1_MPHP_LIVER * AliM);

dt (ABileM) = K1_MPHP_LIVER * (AliM - AliM_lag);

dt (AstM) = Qst * (CA_MPHP - CVstM);

dt (AguM) =

Qgu * (CA_MPHP - CVguM) +

((1 - escapeFrac) * dt (AMgu)) -

(Bowelswitch * K1_MPHP_GUT * AguM) +

(K1_MPHP_LIVER * AliM_lag);

dt (ABowelM) = K1_MPHP_GUT * AguM;

dt (AfaM) = Qfa * (CA_MPHP - CVfaM);

dt (AspdM) = Qspd * (CA_MPHP - CVspdM);

dt (AkiM) = Qki * (CA_MPHP - CVkiM) - K1_MPHP*AkiM;

dt (ArpdM) = Qrpd * (CA_MPHP - CVrpdM) ;

#unbound model

dt (ARBC_MPHP) = (CA_MPHP - CRBC_MPHP / PrbcM);

#dt (ARBC_MPHP) = 0;

dt (Aplasm_MPHP) = QCMC * (CVM - CA_MPHP) - dt (ARBC_MPHP) + (escapeFrac * dt (AMgu));

#********************************************************************************************!

# MPHP Urinary excretion

#********************************************************************************************!

dt (AMMPHPB_MOH) =

dt (AMliM) * FracMetabMOH * (MWMPHP / MWDPHP) * (MWMPHPOH / MWMPHP) -

(K1_MOH * AMMPHPB_MOH);

dt (AMMPHPB_cx) =

dt (AMliM) * FracMetabcx * (MWMPHP / MWDPHP) * (MWMPHPcx / MWMPHP) -

(K1_cx * AMMPHPB_cx);

dt (AMMPHPU_MOH) = K1_MOH * AMMPHPB_MOH;

dt (AMMPHPU_cx) = K1_cx * AMMPHPB_cx;

Curine_MOH = K1_MOH * AMMPHPB_MOH;

Curine_cx = K1_cx * AMMPHPB_cx;

Curine_MPHP = K1_MPHP*AkiM* (MWMPHP / MWDPHP);

}

#These are new addition. Also specified in final four lines of 'outputs'

CalcOutputs

{

CAT_DPHP = Aplasm_DPHP/(MWDPHP*Vplas)*1e6; # Total concentration in plasma (nmol/l)

CARBC_DPHP = ARBC_DPHP / (MWDPHP*VRB)*1e6; # Total concentration in RBC (nmol/l)

CV_total_nmol = (CAT_DPHP + CARBC_DPHP); # Total concentration in blood (nmol/l)

Blood_DPHP = (Aplasm_DPHP + ARBC_DPHP)/(VRB + Vplas); # Total concentration DPHP in blood (mg/l)

Blood_MPHP = (Aplasm_MPHP + ARBC_MPHP)/(VRB + Vplas) * (MWMPHP / MWDPHP) ; # Total concentration MPHP in blood (mg/l)

Blood_cx = AMMPHPB_cx/(VRB + Vplas); # Total concentration of cx in blood (mg/l)

Blood_OH = AMMPHPB_MOH/(VRB + Vplas); # Total concentration of cx in blood (mg/l)

Urine_cx = AMMPHPB_cx*K1_cx; #Rate of deposition of cx in urine (mg/h)

Urine_OH = AMMPHPB_MOH*K1_MOH; #Rate of deposition of OH in urine (mg/h)

Urine_MPHP = K1_MPHP*AkiM * (MWMPHP / MWDPHP); #Rate of deposition of MPHP in urine (mg/h)

}

End.
